# Hydroquinone‐Free, Tetrahexyldecyl Ascorbate Antioxidant Serum for Hyperpigmented and Photodamaged Skin to Achieve Skin Health

**DOI:** 10.1111/jocd.70826

**Published:** 2026-04-07

**Authors:** McKenzie E. Maloney, May Hall, Ryan C. Kelm, Tatiana Kononov, Alisar Zahr

**Affiliations:** ^1^ Department of Internal Medicine Massachusetts General Hospital Boston Massachusetts USA; ^2^ Advanced Dermatology and Skin Surgery Asheville North Carolina USA; ^3^ Clinic 5C Spokane Washington USA; ^4^ Revision Skincareâ Irving Texas USA

**Keywords:** antioxidant, hyperpigmentation, photo‐rejuvenation, ROS, THD ascorbate, vitamin C

## Abstract

**Background:**

Ascorbic acid (AA) has protective and corrective functions critical for counteracting extrinsic and intrinsic skin aging and hyperpigmentation, but it is highly unstable, making it challenging to formulate into skincare products. Tetrahexyldecyl (THD) Ascorbate, a lipid‐soluble derivative of AA, has superior stability and skin‐mimicking properties.

**Aims:**

To investigate the efficacy and tolerability of a novel antioxidant serum containing 30% THD Ascorbate (THD‐AA serum), a patent‐pending blend of antioxidants and prebiotics, on photoaged and hyperpigmented skin with respect to long‐term skin health.

**Methods:**

Using preclinical models and a randomized, double‐blind clinical trial, the antioxidant potential, antimelanogenesis, and antiaging properties of the THD‐AA serum were evaluated.

**Results:**

Using an in vitro tissue model exposed to blue light, there was an 88% reduction in reactive oxygen species (ROS) formation after 30 min, 87% reduction after 60 min, and an 82% reduction after 120 min compared to the blue light‐exposed control. Melanin production was reduced by 24% in vitro tissue co‐culture. THD‐AA serum improved the structural architecture of the skin, including the epidermis, dermal‐epidermal junction, and dermis, and upregulated dermal collagen production 4‐fold compared to a controlled moisturizer in an ex vivo model. In the clinical trial, existing damage and hyperpigmentation were visibly corrected on VISIA‐CR and Antera 3D photographs, as well as in Clinical Grader results. There were no adverse events, and participants tolerated the serum well.

**Conclusions:**

THD‐AA serum has clinical and molecular efficacy in buffering ROS, reducing melanogenesis, and promoting antiaging, providing a safe alternative to hydroquinone products.

**Abbreviations**
AA: ascorbic acid
DEJ: dermal‐epidermal junction
FDA: U.S. food & drug association
HEV: high‐energy visible
OTC: over the counter
ROS: reactive oxygen species
THD: tetrahexyldecyl
THD‐AA Serum: THD ascorbate antioxidant serum

## 1 Introduction

Photodamage accelerates the natural skin aging process, upregulating collagen degradation, downregulating collagen synthesis, and accelerating the production of free radicals. Clinically, photodamage manifests with wrinkling, loss of skin hydration and structure, and dyspigmentation. Undesirable hyperpigmentation, from photodamage and inflammation, affects millions of individuals worldwide, most commonly presenting as solar lentigines, post‐inflammatory hyperpigmentation, and melasma [1, 2, 3]. The management of undesirable hyperpigmentation primarily focuses on skin lightening, as prevention is challenging and often limited to photoprotection [2, 4]. In 2022, the U.S. (FDA) issued warning letters to companies marketing over‐the‐counter (OTC) skin‐lightening products containing undeclared or mislabeled hydroquinone [5]. Because OTC hydroquinone now requires either a new drug application or an approved registration, and prescription hydroquinone remains restricted to specific indications (such as moderate‐to‐severe melasma), a notable gap has emerged for a safe, effective, and widely accessible topical agent for dyspigmentation.

L‐Ascorbic acid (AA), or Vitamin C, is widely used in skincare due to its robust antioxidant capacity, promotion of collagen synthesis, and inhibition of melanogenesis [6, 7, 8]. AA naturally occurs in the skin and serves multiple functions to combat both intrinsic and extrinsic aging [9, 10]. Environmental aggressors, such as urban dust, ozone, ultraviolet radiation, and blue light (440–450 nm), induce the production of ROS and impair barrier function, resulting in outward signs of skin aging (skin tone, skin redness, fine lines, and wrinkles) [11, 12, 13]. By scavenging ROS, AA mitigates oxidative stress, which underlies photo‐aging, inflammation, and hyperpigmentation [14, 15]. Concurrently, AA downregulates tyrosinase activity and melanin polymerization, resulting in skin lightening, and upregulates collagen type I and III synthesis, reducing the appearance of wrinkles [7].

However, topical delivery of AA is constrained by its chemical stability and dermal penetration. AA is a potent antioxidant; thus, it readily oxidizes under ambient conditions, forming its inactive derivative, dehydro‐L‐ascorbic acid, eliminating its antioxidant potential in the skin [7]. Further, given its hydrophilic, ionized state at physiologic pH, it is repelled by the lipophilic stratum corneum [7]. Therefore, some formulations rely on acidifying the skin to temporarily disrupt the barrier and allow some of the formulation to diffuse. To overcome this, more stable AA derivatives have been developed for use in skincare. One promising derivative is tetrahexyldecyl (THD) ascorbate, a lipophilic derivative, which was recently shown to have exponentially superior absorption compared to other derivatives (Maloney et el., under review) [16]. However, there is a dearth of research on this derivative's biological function and clinical results [17]. Therefore, the purpose of this study is to evaluate a 30% THD ascorbate antioxidant serum's (THD‐AA serum) functional properties (antioxidant activity, inhibition of melanogenesis, and collagen production) as well as its clinical efficacy and tolerability.

## 2 Materials and Methods

### 2.1 Antioxidant Activity

To assess the THD‐AA serum's ability to protect against High Energy Visible (HEV) light (450 nm)–induced reactive oxygen species (ROS), an in vitro tissue culture (MatTek EpiDerm, USA) was utilized. Tissues were treated with 100 μL of 5 μM CM‐H2DCFDA, a ROS‐sensitive dye, enabling quantitative measurements of intracellular ROS production. There were three experimental groups: positive control (150 μg/ml Trolox), negative control (untreated), and THD‐AA serum group. The positive control and THD‐AA serum group were exposed to an LED panel composed of 225 blue lights (450 nm, 22 watts) arranged in a square 15x15 matrix for 30, 60, and 120 min. HEV was selected as it causes persistent and prolonged hyperpigmentation and comprises 40%–45% of the atmospheric solar spectrum compared to ultraviolet radiation, which comprises 3%–7% [18].

Mean fluorescence values (RFU) were then determined for each treatment (*n* = 3) at each time point. Data are presented as a percentage of ROS Formation and scaled to 120 min of negative control (Non‐Blue Light exposed tissues). ANOVA and Tukey's multiple comparisons were performed; significance was set to a *p* value less than 0.05.

### 2.2 Melanin Production

To assess the potential of THD‐AA serum to induce changes in tissue pigmentation, an in vitro co‐culture of human keratinocytes and melanocytes (MelanoDerm 3D; MATTEK, USA) was utilized. A saline solution was used as a negative control. Tissues were incubated at 37°C ± 2°C and 5% ± 1% CO_2_. Every 24 h, the tissues were rinsed with saline solution, and fresh material (saline or THD‐AA serum) was applied. Treatments were carried out for 14 days, with four samples per treatment group (*n* = 4). Images were captured with a vanguard inverted scope and a QiClick LED camera using QCapture software at 100× magnification to measure melanin production. Mean treatment values were compared using ANOVA analysis. A *p* value of 0.05 or less was considered significant.

### 2.3 Histopathologic Analysis

Human skin explants from a surgical facelift procedure performed on a 60‐year‐old female subject were utilized for histopathologic analysis. Three experimental groups were included: THD‐AA serum, a positive, and a negative control. THD‐AA is a lipophilic ascorbic acid derivative formed by esterification with fatty alcohol moieties. Thus, for the positive control, hexyldecanol (30%) moisturizer, a common ingredient in cosmeceuticals, was selected because of its lipid‐soluble fatty alcohol structure, which primarily functions as an emollient with some ability to suppress melanin production [19].

Within 4 h of surgical removal, explants were incubated for 4 days with twice‐daily application of controlled moisturizer, THD‐AA serum, and negative control (water) in DMEM/F12 + 10% FBS, on 3 rings of gauze in a 6‐well plate. On the fifth day, tissues were fixed in 10% buffered formalin, processed, and stained with Hematoxylin and Eosin (H&E) and Masson's trichrome stain. Microphotographic documentation was taken with an A12MP BSI back‐illuminated CMOS microscope camera with Sony IMX226 sensor and Amscope software, C‐mounted on an Amscope IN300TC‐FL inverted microscope. Using ImageJ (National Institute of Health, Maryland), the collagen content was quantified and compared across treatments.

### 2.4 Clinical Trial

A randomized, double‐blind, controlled, single‐center study was conducted to compare THD‐AA serum (cell 1) to a 30% hexyldecanol control moisturizer (cell 2) and assess the efficacy and tolerability when used over the course of 12 weeks by women (35–60 years of age) with mild to moderate wrinkles (score 3–6 according to a modified Griffith's scale) and hyperpigmentation on the face. Exclusion criteria included nursing, pregnant or planning to become pregnant individuals, those with allergies to skincare products, those with skin cancer in the past five years, and those who had a rejuvenating procedure (e.g., laser, fillers) in the past six months. Subjects applied the product to a clean face twice daily (AM and PM) along with the provided sunscreen (Neutrogena Ultra Sheer SPF 30, USA) (AM and as needed).

Each subject was clinically graded for fine lines and wrinkles on the crow's feet and global face, skin smoothness (tactile), hyperpigmentation (mottled), hyperpigmentation (discrete), skin tone evenness (redness), radiance, and firmness (visual) on the global face using a modified Griffith's 10‐point scale (0 = none, and 7–9 = severe) at baseline, week 4, 8, and 12 by a PhD trained clinical grader. At baseline, week 8, and week 12, a total of 3 full‐face digital images were taken under lighting conditions, standards, cross‐polarized, and parallel‐polarized using a VISIA‐CR photo‐station (Canfield Imaging Systems) with a Canon Mark II digital SLR camera (Canon Incorporated). Additionally, at baseline, week 8, and week 12, 3D images were captured with Antera 3D Imaging under three filters to focus on surface color, hemoglobin (redness), and melanin (pigmentation). Tolerability was assessed as objective irritation (erythema, edema, and dryness) and subjective irritation (burning, itching, and stinging). Wilcoxon signed‐rank test for comparison from baseline and Wilcoxon rank‐sum test for comparisons between treatment cells were utilized for statistical analysis.

## 3 Results

### 3.1 Antioxidant Activity

**FIGURE 1:**
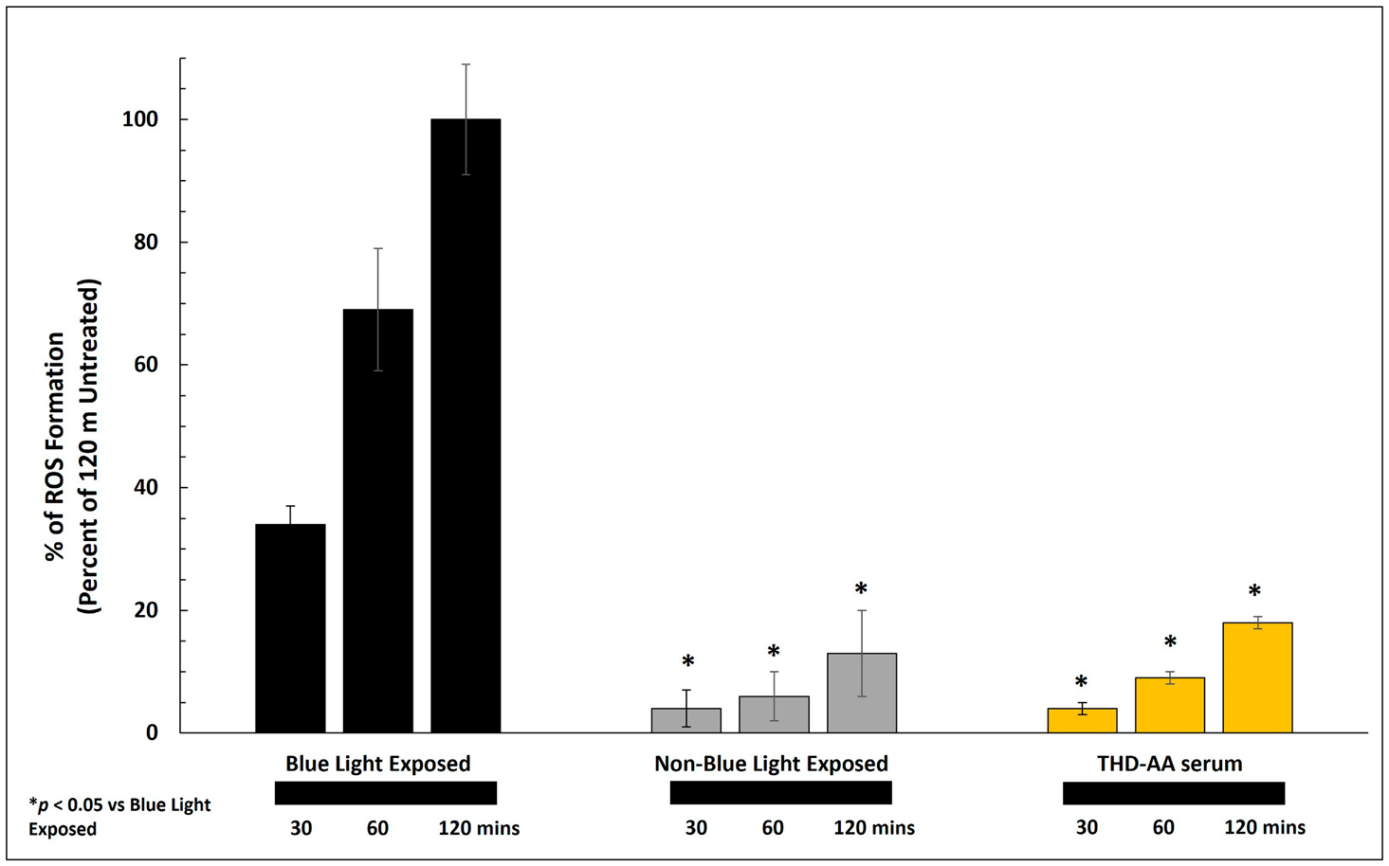
Antioxidant activity of THD ascorbate antioxidant serum when exposed to blue light compared to controls over time.

The THD‐AA serum reduced ROS formation by 88% after 30 min, 87% after 60 min, and 82% after 120 min. Compared to the blue light‐exposed control, THD‐AA serum provided statistically significant (*p* < 0.05) protection from HEV‐induced ROS at all time points (Figure 1). The THD‐AA serum buffered the effect of blue light such that the level of ROS production was not statistically different from the non‐blue light control group at all time points (*p* > 0.05).

### 3.2 Melanin Production

**FIGURE 2:**
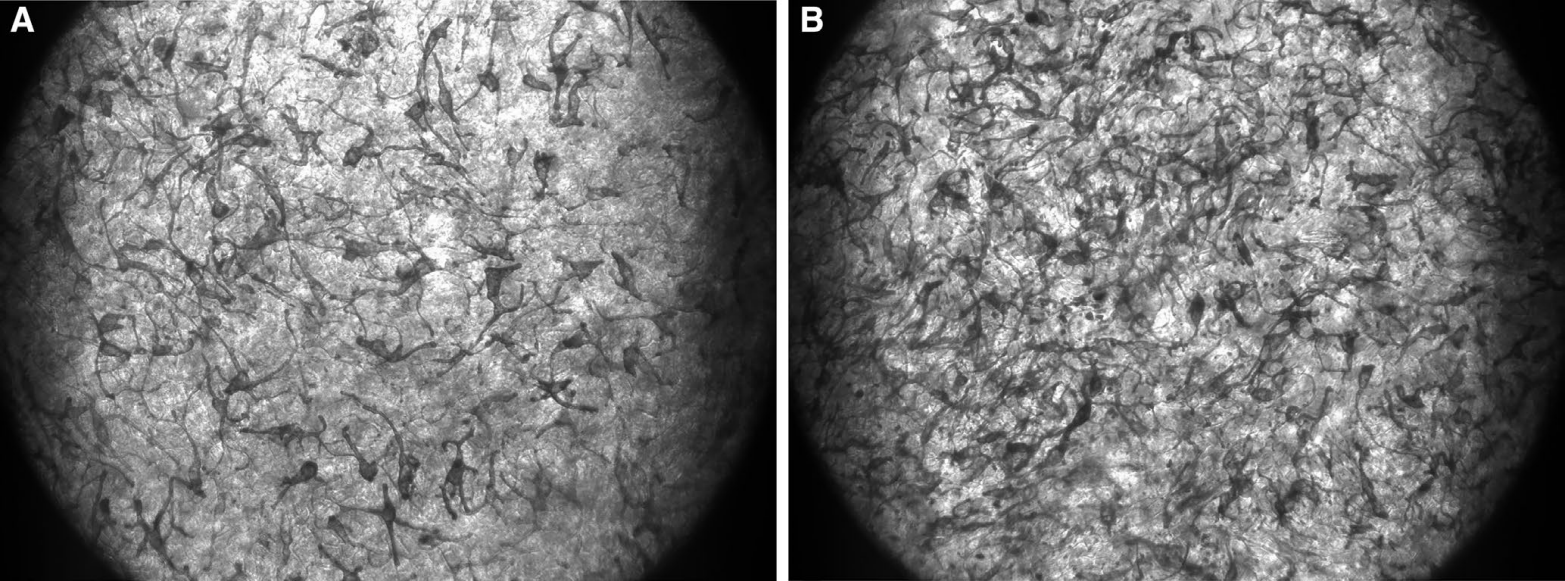
Representative microscopic photos highlighting the statistically significant reduction in melanin production with (A) U (B) untreated samples (*p* < 0.001) captured at day 14, at 100×.

The THD‐AA serum resulted in a statistically significant (*p* < 0.001) decrease in melanin production after 14 days compared with the untreated group. After two weeks, the THD‐AA serum group produced 22.5 μg/tissue compared to 29.5 μg/tissue of melanin in the untreated tissue, representing a 24% reduction in melanin production. Microscopic images of treated tissue at day 14 revealed a decrease in melanocytes compared to untreated tissue (Figure 2).

### 3.3 Histopathologic Analysis

**FIGURE 3:**
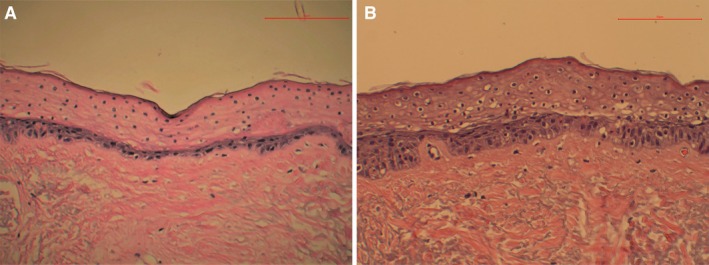
Hematoxylin and eosin (H&E) staining for the positve control moisturizer (A) showed atrophic epidermis featuring a loss of the stratified structure, enucleated cells, and a viable basal layer. The antioxidant serum (B) treated skin presented fewer enucleated cells in the epidermis than the control; however, a multilayered stratum basale was visible, as well as a pronounced dermal‐epidermal junction (DEJ). Images captured at 20×, scale bar indicates 50 μm.

On H&E staining of the positive control, an atrophic epidermis was observed, featuring a loss of the stratified structure, enucleated cells, and a viable basal layer. The THD‐AA serum‐treated skin presented with fewer enucleated cells in the epidermis than the control; however, a multilayered stratum basale was visible, as well as a pronounced dermal‐epidermal junction (DEJ) (Figure 3).

**FIGURE 4:**
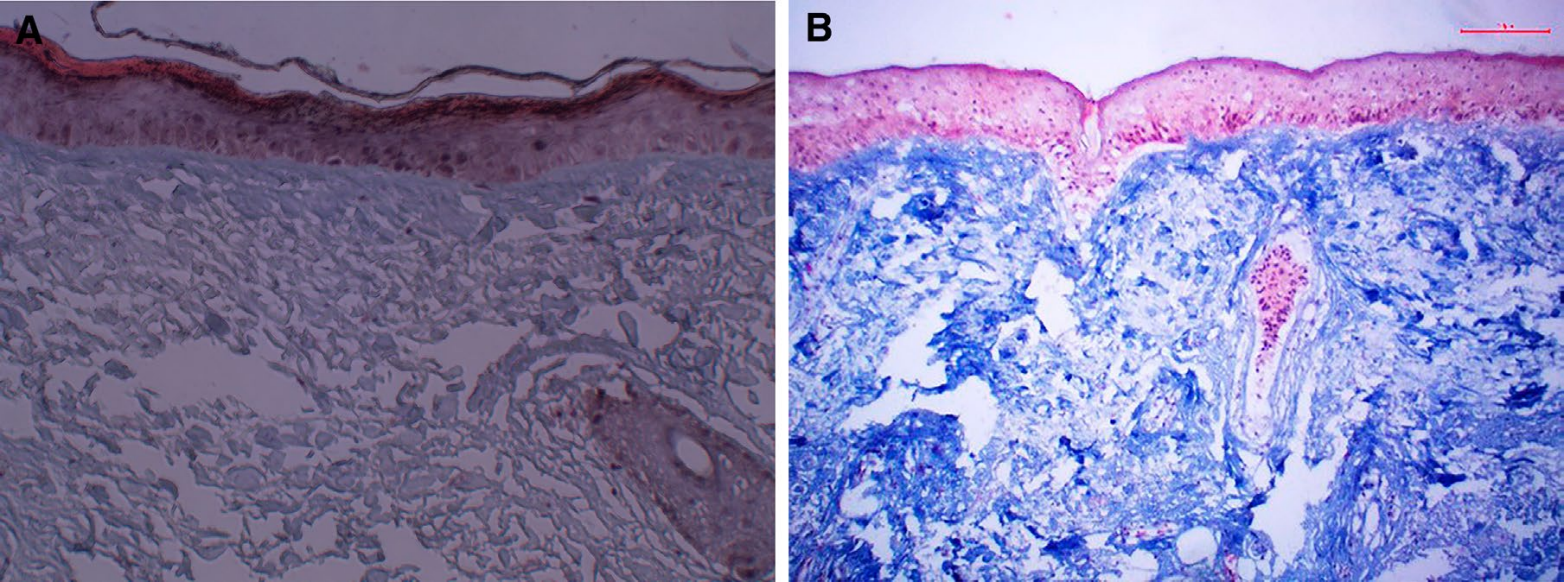
Masson's trichome stain was performed on tissues treated with (A) positive control moisturizer and (B) the THD ascorbate antioxidant serum. Images captured at 20×.

To evaluate collagen production, Masson's trichrome stain was performed (Figure 4). Measurements of color intensity performed on images (*n* = 3) revealed a 4‐fold increase of collagen in the THD‐AA serum compared to control‐treated tissue.

### 3.4 Clinical Trial

**FIGURE 5:**
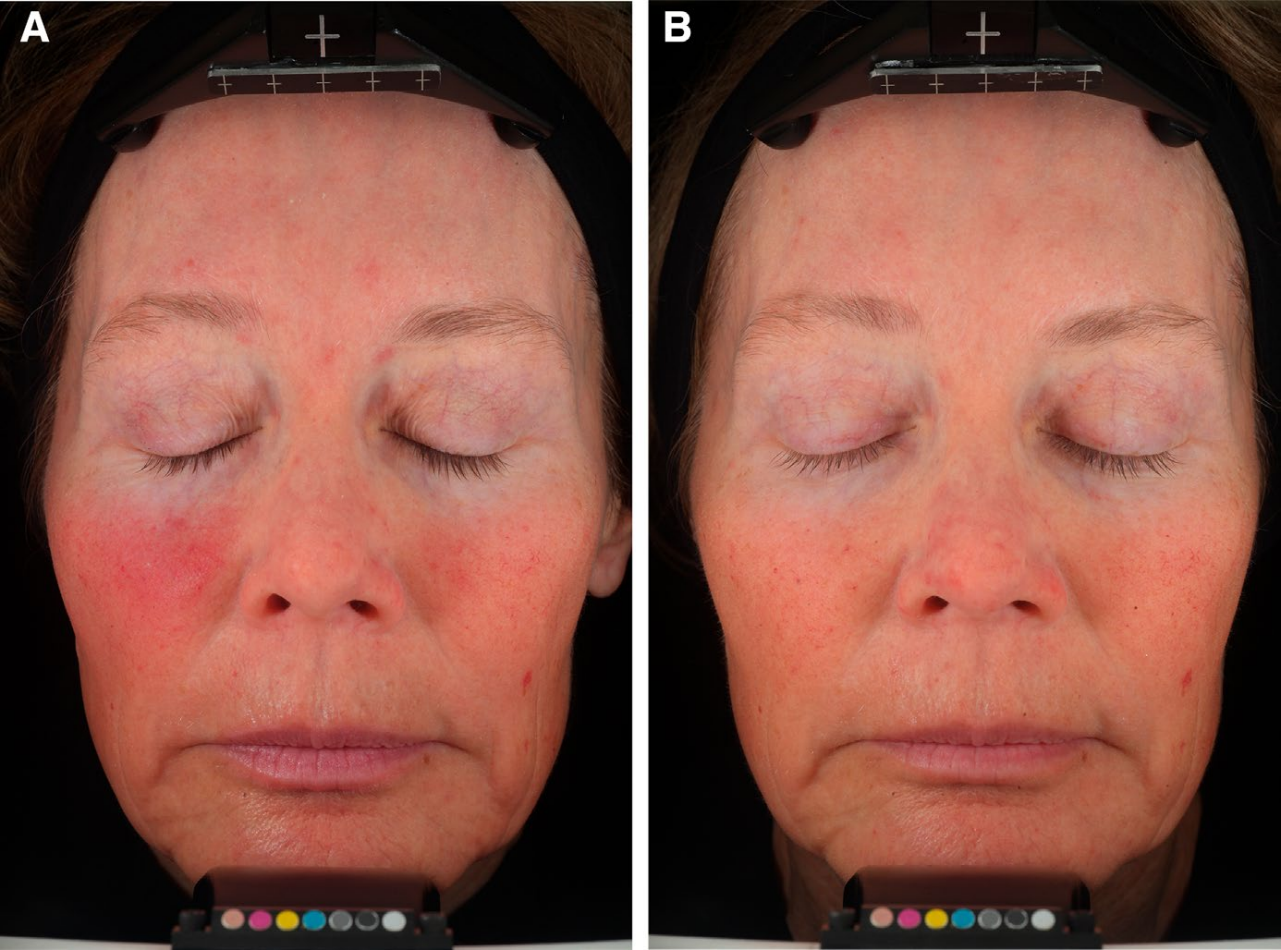
A representative subject, 57 years of age with Fitzpatrick Skin Type III, shown at (A) baseline and (B) 12 weeks using VISIA‐CR image with cross‐polarized light.

**FIGURE 6:**
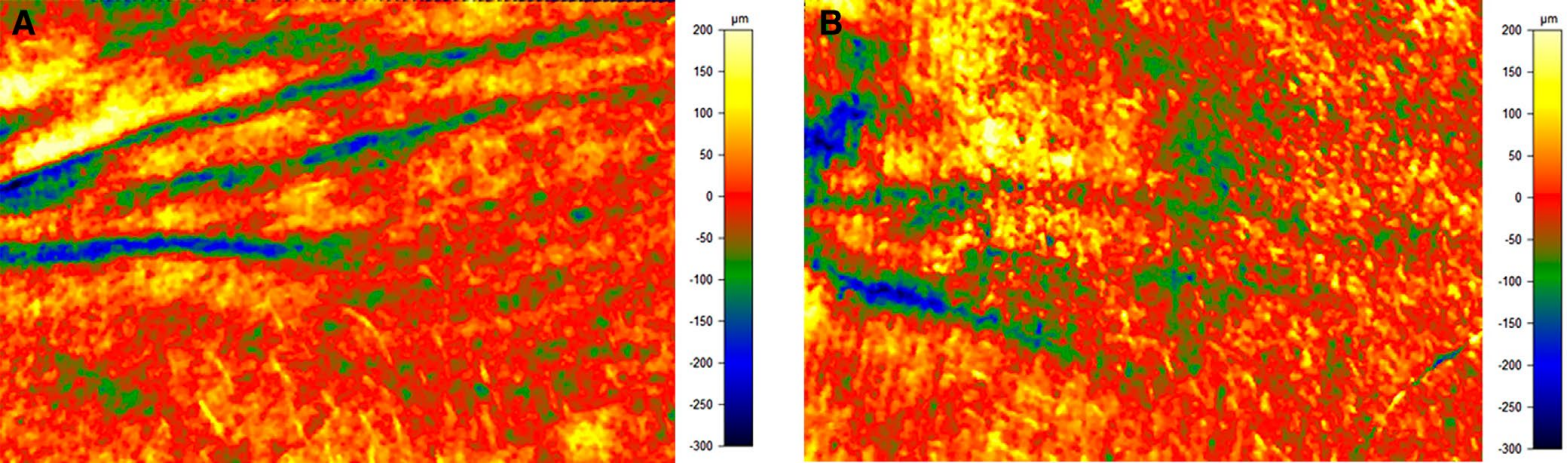
Reduction in Wrinkle Volume and Depth as measured by PRIMOS lite. Images taken of crow's feet area at (A) baseline and (B) week 12.

Sixty‐two subjects completed the clinical trial, and thirty‐one subjects were randomized to each group. The average age of participants was 52 (range 38–60), and most were Fitzpatrick Skin Type III (56.5%). As early as 4 weeks, treatment comparisons from baseline analysis indicate the THD‐AA serum outperformed the control for radiance, fine lines (crow's feet and global), skin tone evenness (photodamage and hyperpigmentation), and skin smoothness. By week 8, 88% of subjects showed an improvement in melanin. Representative baseline and week 12 photos are shown in (Figure 5). At week 12 the following statistics were found in the THD‐AA serum group:

- 87% of participants showed an improvement in the appearance of facial lines.
- 87% of participants showed an improvement in the appearance of skin smoothness.
- 68% of participants showed an improvement in the appearance of skin radiance.
- 77% of clinical study participants showed a decrease in wrinkle volume (10% average reduction), and 73% of clinical study participants showed a decrease in wrinkle depth (10% average reduction) (Figure 6).

The results from the subjective self‐assessment conducted at week 12 were as follows:

- 87% of users reported visibly brighter skin.
- 94% of users reported an improvement in the evenness of skin tone.
- 90% of users reported an improvement in overall skin appearance
- 84% of users reported visibly firmer skin
- 90% of users agreed overall the product was mild and did not irritate my skin

There were no significant changes from baseline for any tolerability parameter (burning, itching, or stinging).

## 4 Discussion

The present study evaluated a novel 30% THD‐AA serum incorporating a synergistic blend of lipophilic and hydrophilic antioxidants (acetyl zingerone, *Plantago lanceolata* leaf extract, and hydrolyzed *Eruca sativa* extract) in a prebiotic oil‐in‐water emulsion containing sodium carboxymethyl β‐glucan. Together, these constituents were engineered to enhance THD ascorbate's molecular stability and cutaneous penetration and improve skin‐barrier resilience. In vitro, ex vivo, and clinical findings presented here collectively demonstrated that this formulation provides broad protection from oxidative and environmental stress, suppresses melanogenesis, and stimulates dermal remodeling, translating to measurable clinical improvement in photodamaged and hyperpigmented skin.

Vitamin C is a well‐established antioxidant in skin; however, its clinical performance has historically been limited by rapid oxidation and poor dermal bioavailability [7, 10, 15, 20]. Traditional topical vitamin C derivatives are constrained by the need for acidic environments to penetrate the skin [7]. THD ascorbate overcomes these barriers through its lipid solubility, enabling diffusion across the stratum corneum and conversion to active ascorbic acid within the epidermis and dermis, exponentially outperforming other hydrophilic derivatives in terms of penetration capabilities (Maloney et al., under review) [21]. Consistent with prior literature [20, 22, 23], THD‐AA serum demonstrated robust protection against oxidative stress, inhibiting ROS production induced by high‐energy visible light by 82%. This represents a 22%–42% enhancement in antioxidant activity compared to other derivatives, likely a result of its superior skin penetration [20, 22, 23].

Vitamin C's antioxidant potential not only underlies its anti‐aging and anti‐carcinogenesis properties but also its anti‐melanogenesis properties [24, 25]. With photodamage, melanogenesis is upregulated in an effort to absorb UV radiation and UV‐generated ROS [26]. To combat this in melanocytes, Vitamin C quenches ROS, downregulating melanogenic cascades [24, 25]. Additionally, Vitamin C acidifies the cytoplasm, inhibiting the tyrosinase protein, the rate‐limiting step in melanogenesis [22, 27]. In this study, THD‐AA serum reduced melanin production by 24% in the MelanoDerm tissue model, demonstrating potent anti‐melanogenic activity. Clinically, this results in reduced hyperpigmentation, as demonstrated by 88% of participants showing improvement by week 8, which is consistent with prior studies. A previous prospective, open‐label pilot study with 10 female subjects with melasma evaluated the efficacy of twice‐daily 30% THD ascorbate for 12 weeks during the summer. All subjects experienced a decrease in hyperpigmentation with an average improvement of 33.7% [14]. THD ascorbate was also evaluated in combination with 0.05% retinol to treat mild‐to‐moderate hyperpigmented and photodamaged skin. Forty‐four female subjects applied 30% THD ascorbate daily and 0.05% retinol every other night for two weeks before advancing to nightly retinol application for a total of 12 weeks. After 12 weeks, 75% experienced a reduction in global hyperpigmentation by an average improvement of 9.9%, and 77% with discrete hyperpigmentation with an average improvement of 11% [8].

THD‐AA serum's ability to reduce melanogenesis is clinically meaningful given the changing therapeutic landscape for pigmentary disorders. In 2022, the U.S. FDA withdrew authorization for over‐the‐counter hydroquinone products, citing safety concerns, including exogenous ochronosis and irritant dermatitis [5]. As a result, dermatologists and patients face limited non‐prescription options for addressing melasma, post‐inflammatory hyperpigmentation, and photo‐induced dyspigmentation [4, 28]. Within this context, a stable, topical, non‐phenolic, non‐cytotoxic antioxidant capable of down‐regulating melanogenesis represents a timely and clinically relevant alternative. The current formulation's dual antioxidant and pigment‐modulating actions satisfy both efficacy and safety criteria, supporting its potential role as a frontline topical brightening agent.

Regarding THD‐AA serum's neocollagenesis, this study's ex vivo analysis revealed a 4‐fold increase in collagen production compared with control, accompanied by improvements in epidermal, dermal, and dermal‐epidermal junction architecture. These findings are consistent with vitamin C's function as a cofactor for prolyl and lysyl hydroxylases, enzymes essential for collagen maturation and cross‐linking [29]. Vitamin C also suppresses matrix metalloproteinases (MMP‐1 and MMP‐9), thereby preserving extracellular matrix integrity [16]. Clinical evaluation confirmed that the formulation visibly corrected existing photodamage, as assessed by VISIA‐CR and Antera 3D imaging, investigator and subject grading. On average, participants experienced a 10% reduction in wrinkle volume and depth, as well as improvements in overall brightness, pigmentation uniformity, fine lines, and surface texture. Subjects reported favorable sensory attributes and sustained hydration, and the pH‐neutral formulation was well tolerated, with no reports of erythema, stinging, or barrier disruption.

In the present formulation, THD ascorbate was complemented by an additional antioxidant blend and prebiotic system, providing additive and potentially synergistic benefits. Acetyl zingerone neutralizes ROS, inhibits advanced glycation end‐product formation, and stabilizes THD ascorbate, increasing its bioavailability [30]; *Plantago lanceolata* extract reduces ROS and inflammatory cytokine production [31]; and the prebiotic, β‐glucan, enhances epidermal recovery and hydration while influencing the skin microbiome [32]. This multifactorial approach provides continuous defense against both intrinsic and extrinsic aging pathways, an advantage over single‐molecule antioxidants that act transiently and degrade rapidly. Further, the THD ascorbate antioxidant serum reduces chronic inflammation, improves intercellular communication, and reduces extracellular matrix changes, which represent an entry point for buffering against all the hallmarks of aging and promoting long‐term skin health [33].

A modest sample size limited these studies; in particular the ex vivo study to measure the mechanism of action had a sample size of one. However, despite the modest sample sizes, there was sufficient power to detect a statistically significant difference between the treatment groups. Additionally, the clinical trial was limited by the diversity of participants, with the majority classified as Fitzpatrick Skin Type III. Future clinical trials should continue to recruit diverse patient populations to reflect the general population, and direct comparisons with other depigmenting agents (e.g., 4% hydroquinone, retinoid combinations) would further substantiate the clinical utility.

## 5 Conclusion

This study demonstrated that a 30% THD‐AA serum provided potent antioxidant protection, significant inhibition of melanogenesis, and robust stimulation of collagen synthesis, resulting in clinically significant results. The formulation's pH‐neutral, prebiotic‐enhanced emulsion ensures stability, penetration, and tolerability, translating mechanistic benefits into visible clinical improvement. In an era where hydroquinone restrictions have highlighted the need for safe and effective alternatives, this lipophilic vitamin C system represents a scientifically validated, well‐tolerated option for the prevention and correction of photoaging and pigmentary disorders.

## Author Contributions

McKenzie E. Maloney drafted and edited the manuscript. May Hall and Ryan C. Kelm critically reviewed the manuscript. Tatiana Kononov and Alisar Zahr designed and conducted the experiments. All authors reviewed and approved the final version of the manuscript.

## Funding

This work was sponsored by Revision Skincare LLC.

## Ethics Statement

This study was conducted in accordance with all applicable guidelines for the protection of human subjects for research as outlined in 21 CFR 50, the accepted standards for Good Clinical Practice (GCP), and the standard practices of Stephens & Associates.

## Consent

All patients provided informed consent for the use of their clinical information and/or images for research and publication purposes.

## Conflicts of Interest

McKenzie E. Maloney and Tatiana Kononov are consultants at Revision Skincare. May Hall and Ryan C. Kelm are board‐certified dermatologists and key opinion leaders in vitamin C technology. Alisar Zahr is a full‐time Revision Skincare employee.

## Data Availability

Data available on request from the authors.
