# Correction to “Hydroquinone‐Free, Tetrahexyldecyl Ascorbate Antioxidant Serum for Hyperpigmented and Photodamaged Skin to Achieve Skin Health”

**DOI:** 10.1111/jocd.70895

**Published:** 2026-05-21

M. E. Maloney, M. Hall, R. C. Kelm, T. Kononov, A. Zahr, “Hydroquinone‐Free, Tetrahexyldecyl Ascorbate Antioxidant Serum for Hyperpigmented and Photodamaged Skin to Achieve Skin Health,” *Journal of Cosmetic Dermatology* 25, no. 4 (2026): e70826. https://doi.org/10.1111/jocd.70826.

In the caption of Figure 2, some inaccuracies were identified. The corrected caption, along with the figure, is provided below.

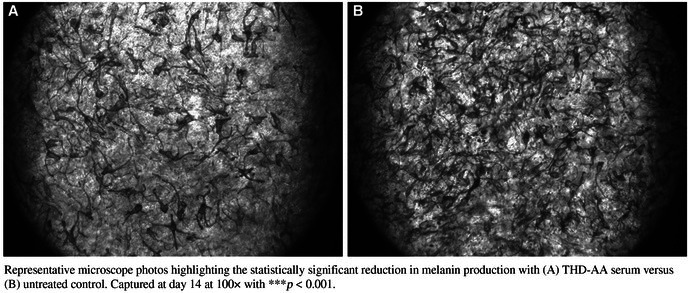

We apologize for this error.

